# Influence of changing patterns in lung cancer treatment and survival on the cost-effectiveness of CT screening: a modeling study

**DOI:** 10.1016/j.eclinm.2025.103446

**Published:** 2025-08-29

**Authors:** Koen de Nijs, Kevin ten Haaf, Dana Moldovanu, Juul Hubert, Isabelle van den Bosch, Anouk Eijkelboom, Carlijn van der Aalst, Harry J. de Koning

**Affiliations:** a Department of Public Health, Erasmus MC - University Medical Center Rotterdam, Rotterdam, 3015 CE, the Netherlands; b Department of Research and Development, Netherlands Comprehensive Cancer Organisation, Rijnkade 5, Utrecht, 3511 CV, the Netherlands

**Keywords:** Lung neoplasms, Immunotherapy, Molecular targeted therapy, Tomography, X-ray computed, Mass screening, Cost-effectiveness analysis

## Abstract

**Background:**

With the introduction of immune- and targeted therapies, lung cancer survival has lengthened, but per-patient costs of treatment have also increased. Both the clinical outcomes and costs of late stage disease have bearing on the benefits and relative cost of early detection interventions. Cost-effectiveness estimates of lung cancer (LC) computed tomography (CT) screening, crucial for policymaking, using such real-world recent data have been limited.

**Methods:**

Registry data of the entire Dutch LC patient population (n = 137,129) inform treatment cost and real-world survival before (2012–2017) and after (2018–2021) widespread novel therapy introduction. The MISCAN-Lung (MIcrosimulation SCreening Analysis) microsimulation model projects the population-level benefits and harms of CT screening for Dutch 1949–1979 cohorts.

**Findings:**

From 2012–2017 to 2018–2021, per-patient care expenditures increased 52%. Survival improvements differ by patient subgroup; for males <65 y, 3-year relative survival for stage-IV adenocarcinoma increased from 10.6% to 22%. MISCAN model simulations found annual screening ages 55–75 from 1.51% PLCOm-risk (Prostatem Lung Colorectal Ovarian Screening trial model) as cost-effective (<€20,000 per Quality Adjusted Life Years Gained (QALYG)). After adjusting LC survival to novel therapies, screening is expected to yield 3253 QALYG and 4118 LYG per 100,000 population, 3.2% (QALYG) and 3.7% (LYG) lower than before novel therapies. However, expected net screening costs decrease 16.7% as late-stage treatment has become more expensive and is applied longer; the savings in late-stage therapy cost were estimated to have increased 183%. The cost per QALYG decreased 13.9%, from €14,172/QALY to €12,201/QALY.

**Interpretation:**

Novel treatments for late-stage lung cancer have made lung cancer screening more cost-effective. While LC survival improves due to novel treatments for advanced disease, the increased expenditures outpace survival gains. Screening implementation still needs prioritization, even as late-stage LC survival improves.

**Funding:**

European Union Horizon 2020 grant 848294: 4-IN-THE-LUNG-RUN. VENI grant number 09150161910060 (10.13039/501100003246Dutch Research Council/10.13039/501100001826Netherlands Organisation of Health Research (ZonMW)).

**Research in context**

**Evidence before this study**

We reviewed previous evidence by searching PubMed for all literature that discussed lung cancer, screening, as well as novel therapies. Specifically, we used the search string: *(“Lung Neoplasms”[MeSH] OR “lung cancer”[Title]) AND (“Mass Screening”[MeSH] OR “Early Detection of Cancer”[MeSH] OR “lung cancer screening”[Title] OR “low-dose CT”[Title/Abstract] OR “LDCT”[Title/Abstract]) AND (“Immunotherapy”[MeSH] OR “Antineoplastic Agents, Immunological”[MeSH] OR “Molecular Targeted Therapy”[MeSH] OR “targeted therapy”[Title/Abstract] OR “immunotherapy”[Title/Abstract] OR “immune checkpoint inhibitor”[Title/Abstract] OR “nivolumab”[Title/Abstract] OR “pembrolizumab”[Title/Abstract] OR “EGFR inhibitor”[Title/Abstract] OR “tyrosine kinase inhibitor”[Title/Abstract])*. This returned 119 articles, of which 5 articles performed cost-effectiveness analysis of lung cancer screening incorporating recent therapy effectiveness. None of the studies used registry data to evaluate real-world survival under novel therapy use, nor were the data underlying the effectiveness and the cost of the treatment obtained from the same source.

**Added value of this study**

We add to the literature by evaluating cost and survival of lung cancer under novel treatment regimens using real-world data from a cancer registry with national coverage, and propagating those results onto scenarios of lung cancer screening cost-effectiveness.

**Implications of all the available evidence**

Policy makers and clinicians should continue to prioritize targeted lung cancer screening, which was found be more cost-effective under novel treatment regimens. High late-stage costs relative to modest improvements in survival mean that early detection is more cost-effective than before.

## Introduction

In Europe, lung cancer is the fourth most common cause of death in men and fifth in women, first of all cancers.^1^ It is associated with 226 thousand deaths annually, 4.3% of all deaths.^2^ To combat this source of mortality and morbidity, the thoracic oncology field has seen the onset of two major developments: the increased prescription of targeted- and immunotherapies, as well as the uptake of computed tomography screening.^3^ Whereas the former improves outcomes for those diagnosed with advanced-stage lung cancer, the latter aims to improve outcomes by advancing detection of the cancer to early-stage, surgically resectable disease.

In the National Lung Screening Trial (NLST) and Dutch-Belgian Lung Cancer Screening Trial (NELSON), regular Computed Tomography (CT) screening was demonstrated to reduce lung cancer mortality by 20 and 24%, respectively.^4^^,^^5^ In light of positive trial results, the European Commission issued a positive recommendation of lung cancer screening among individuals identified as being at high risk of lung cancer based on at least the age and smoking history.^6^

Meanwhile, the lung cancer treatment pathway for those with clinically detected lung cancer is becoming populated with novel therapies. Immunotherapies and targeted therapies, are increasingly used for those with stage III–IV lung cancer, and are also seeing uptake in stage II disease.^7^ In clinical trials, as well as in clinical practice, these novel therapies have demonstrated increased survival.^8^, ^9^, ^10^ In the Netherlands, median survival for metastatic non-small cell lung cancer has increased by two months between 2000 and 2022.^11^ They are also known to carry a significant financial burden.^12^ Their introduction increased the treatment cost per Dutch lung cancer patient by 52% (net of inflation) in the 5 years from 2016 to 2021.^7^

These two interventions: novel treatments and CT screening, both aim to improve outcomes for those with lung cancer. Because early detection prevents late-stage incidence, the cost-effectiveness of interventions aimed at early detection is interlinked with the cost and prognosis of late-stage disease. However, current estimates of the cost-effectiveness of CT screening for lung cancer often use treatment costs from analyses prior to the implementation of these novel treatments,^13^, ^14^, ^15^ warranting renewed evaluation of the cost-effectiveness of screening in the novel treatment landscape.

Recent cost-effectiveness estimates of lung cancer screening for Australia compare contemporary treatment regimens to the cost and survival of previous regimens.^16^ They found the cost-effectiveness of screening to be 4% improved due to a 14.8% reduction in net cost relative to 11.3% fewer life years gained. However, their period-specific survival estimates are inferred from survival hazards obtained from the trial setting. Treatment efficacy is known to differ between real-world and clinical trial settings.^17^ Here, we leveraged cancer registry data to ascertain cost and survival pre- and post the introduction of novel therapies, using the entire Dutch lung cancer patient population (n = 137,129).^7^ Using these estimates, we then performed a cost-effectiveness analysis of CT lung cancer screening using the MIcrosimulation SCreening ANalysis (MISCAN)-Lung microsimulation model, studying particularly the effect of varying the treatment cost, as well as survival after diagnosis, from before to after the introduction of novel therapies.

## Methods

We perform a health economic evaluation of lung cancer screening for the Dutch population, with particular attention to the sensitivity of the cost-effectiveness estimate to novel treatment patterns.

First, cost and survival estimates were obtained from the Dutch cancer registry, specific to periods before (prior to 2018) and after (2018 and later) widespread introduction of targeted- and immunotherapies. After the approval of pembrolizumab in 2017, immune- and targeted therapy use has been seen to increase sharply from 2018 onwards for metastatic Non Small Cell Lung Cancer (NSCLC).^7^^,^^11^

Second, these were applied to the incidence and staging patterns generated by MISCAN-Lung in a screening scenario and a no-screening scenario. Primary outcome measures include the net cost of the screening scenario relative to no screening, as well as the life years (LY) and Quality-Adjusted Life Years (QALYs) gained relative to no screening.

### Statistical analysis

#### Modeling of screening effectiveness

The MISCAN-Lung model is a microsimulation model of lung cancer natural history. The model simulates complete individual life histories from birth, including smoking initiation or cessation, lung cancer onset, cancer detection and subsequent survival and smoking-related non-lung cancer mortality. Outcomes from individual life histories are aggregated to give population-level forecasts of lung cancer burden, and estimates of the effectiveness of interventions such as CT screening or smoking cessation interventions. This allows extrapolation of trial-based effectiveness of an intervention to population-wide and lifetime perspectives. The model has previously informed (cost) effectiveness estimates of lung cancer screening for the US, Switzerland, Ontario, Australia, and the Netherlands.^13^, ^14^, ^15^^,^^18^, ^19^, ^20^, ^21^ An exhaustive model description is given in the methodological supplement, as well as in previous publications.^15^^,^^19^ We assume a public payer perspective. Health economic assumptions are gathered in the supplemented Consolidated Health Economic Evaluation Reporting Standards (CHEERS) checklist.^22^

#### Screening scenarios

The MISCAN model may also simulate lung cancer screening per a specified screening strategy, accounting for the age and smoking history of the individual to determine eligibility for screening, maintaining either packyear-based eligibility criteria or a reduced form PLCOm2012 risk model, as employed in the UK Targeted Lung Health Check.^23^^,^^24^ If a screening event is simulated, any preclinical cancer may be detected, with the CT sensitivity specific to the stage and histology of the cancer at the time of the screen. If the cancer is detected, it may be cured with a probability specific to the cancer stage. If not cured, the time of lung cancer death of the scenario without screening is maintained. Parameters of screening effectiveness were calibrated to individual-level data from the Dutch-Belgian NELSON screening trial. Current implementation efforts of lung cancer screening vary by the screening strategy employed. We therefore use a few exemplary strategies to test the cost-effectiveness of a potential lung cancer screening programme.

• A-TLHC: Screening per the UK Targeted Lung Health Check criteria of minimum 1.51% PLCOm^24^ risk between ages 55 and 75 (A-TLHC), but with an annual interval.^25^
• B-TLHC: Biennial screening per the UK TLHC.^23^
• A-USPSTF: Annual screening per United States Preventive Services Task Force (USPSTF) 2021 eligibility criteria of minimum 20 packyears smoked and maximally 15 years since smoking cessation.
• B-USPSTF: Biennial screening with USPSTF eligibility (B-USPSTF).

Although both the USPSTF and TLHC employ annual screening, we include biennial screening as some countries are considering biennial screening for the sake of capacity management.^15^^,^^16^ Perfect adherence to screening is assumed to present results representative for the attending participant.^26^ However, a sensitivity analysis with reduced adherence of 50% is included.

#### Population characteristics

We simulate life histories for individuals born 1945–1979 in the Netherlands. Birth cohort sizes are adjusted to reflect the population composition by sex and birth cohort of the 1st of January 2023. Smoking histories are simulated with probabilities of smoking initiation, smoking cessation, and smoking intensity (measured in Cigarettes per Day [CPD]) set to concur with self-reported smoking behavior from the Dutch Health Survey from 1989 to 2020, by sex and birth-cohort.^19^^,^^27^ A more complete description of simulated smoking behaviors is reported in the methodological supplement. To simulate mortality of causes other than lung cancer, cohort life tables are taken from Statistics Netherlands and adjusted for the relative risk of current or former smokers relative to never smokers.^19^^,^^28^

### Costs and health utilities

Supplementary Table S1 shows the cost and utility values applied to the simulated outcomes. Quality of Life values for lung cancer patients are taken from a meta-analysis of lung cancer health utilities.^29^ Population-level EQ-5D norm utilities by age category and sex are taken from the EuroQoL study.^30^ These represent the average experienced health-related quality of life for individuals of a given age for the Netherlands, as not to assume life years gained at an old age to be lived in perfect health. For simulated individuals with lung cancer, we apply disutility experienced in lung cancer care as a proportion of the norm utility to account for variations by age.^31^

Costs of lung cancer care are obtained by linking individual incident lung cancer cases from the Dutch Cancer Registry with health insurance claims data from 2013 to 2020.^7^ With near-perfect coverage for both the individual-level claims data and the Dutch Cancer Registry, the cost estimates can be considered exhaustive relative to the Dutch population undergoing lung cancer care. Incremental costs per month for initial treatment (first 6 months), terminal-phase treatment (final 6 months), and continuing care (intermittent period) are calculated. The methods and results of this costing study have recently been published,^7^ and are also reported in the methodological supplement. CT cost is taken from the guideline for economic evaluations of the Dutch Health Care Institute.^32^^,^^33^ Administrative overhead per screening participant is adapted from realised costs of the Dutch colorectal cancer screening programme.^34^ Cost, LY and QALYs are discounted 3%,^35^ except in specific sensitivity analyses of the discount rate. Univariate sensitivity analyses are applied to the CT screening cost, as well as stage I–II and stage III–IV cancer treatment costs.

#### Survival estimates

From the same patient records that informed the treatment estimates, we derive relative survival curves. Incident lung cancer cases from 2012 to 2021 are included, with follow-up of survival until the 1st of February 2023. The monthly relative survival is calculated using the Ederer-II method,^36^^,^^37^ adjusting the observed survival to the contemporary expected survival reported by Statistics Netherlands.^38^ Survival estimates are stratified by age at diagnosis (<65, 65–75, 75+), biological sex, stage of cancer (IA, IB, II, IIIA, IIIB, IV) and histology (Adenocarcinoma, Squamous Cell Lung Cancer, Other NSCLC [including unknown histology], Small Cell Lung Cancer). Results are generated by period, with separate estimates for patient-years in the 2012–2017 period, and patient-years lived in the 2018–2023 period. To smoothen the relative survival data, a generalized linear model is fitted to each subgroup-specific curve, assuming a Poisson survival rate. The resulting period-specific survival curves are applied to the life histories of individuals with lung cancer generated in the MISCAN model to generate estimates of the lung cancer mortality rates in a screening and no-screening scenario, before and after the introduction of novel therapies.

### Ethics

No identifiable information was used; therefore, no institutional review board (IRB) approval was needed.

### Role of the funding source

Our funding source had no role in the study design, nor in data collection, analysis and interpretation, nor the writing of the manuscript. All authors had full access to the data and carry the responsibility to submit for publication.

## Results

We first present the shift in treatment patterns towards targeted- and immunotherapies in the Netherlands, as well as their effect on the observed survival and cost patterns. We then apply the resulting period-specific estimates of lung cancer survival and treatment costs to generate outputs from the MISCAN-Lung model. This covers the monetary cost and (quality-adjusted) life years gained from screening, before and after the introduction of novel therapies. Finally, we show sensitivity analyses to test the robustness of our results to further changes to the cost of treatment and screening.

### Effect of novel treatment patterns on survival and costs

First-line treatment for stage III–IV lung cancer was largely dominated by chemo- and radiotherapy in the 2012–2017 period (Supplementary Table S2), with 65% of patients aged below 65 receiving some combination of chemo- and/or radiotherapy, and 24.0% receiving no treatment. For the 2018–2021 period, targeted- and immunotherapy became more prevalent. The share of stage III–IV patients aged below 65 receiving any targeted- or immunotherapy increased from 5.9% in 2012–2017 to 37.9% in 2018–2021. For older patients, the increase was 4.2–28.8% (ages 65–75), and 3.2–15.9% (ages 75+). For stage I–II lung cancer, the dominant treatment remained surgery, covering 65.6% of patients aged below 65 in 2012–2017 to 60.9% in 2018–2021. Treatment beyond the first line is not recorded, and may also include systemic therapies for stage I–II cancers that progress after initial treatment. Although their cost would be included in our costing study (which includes all medical care until death), it is unknown which share of early-stage cancers receive novel therapies beyond progression. As such, novel therapies could be increasing the cost and survival for continuing or terminal-phase care in early-stage as well as in metastatic disease.

**Fig. 1:**
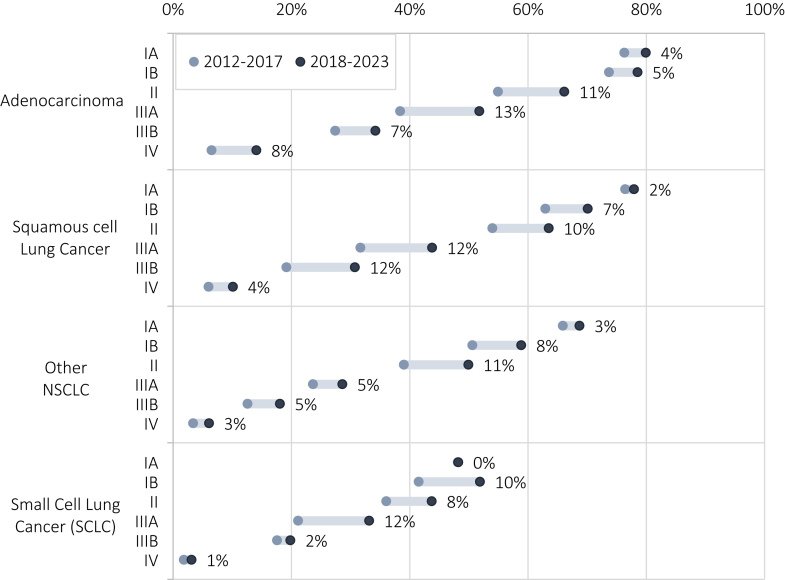
**Cumulative 3-year relative survival of male lung cancer patients by stage, histology and period of incidence, and percentage point increase over the periods.** Figure 1 shows the relative survival of male lung cancer patients aged [65–75) at diagnosis, stratified by the stage, histology, and period of lung cancer treatment. Data labels report the percentage point increase relative to 2012–2017. Relative survival was estimated using the Ederer-II method, after which a Poisson curve was fitted using Generalized Linear Modeling of the monthly relative survival for each patient group (stratified by age at diagnosis [<65, 65–75, 75+], sex, stage of cancer [IA, IB, II, IIIA, IIIB, IV] and histology [Adenocarcinoma, Squamous Cell Lung Cancer, Other NSCLC, Small Cell Lung Cancer]). Period-specific estimates were generated by allocating the person-years of lung cancer patients diagnosed [2012, 2021] to separate 2012–2017 and 2018–2023 periods. Survival curves for other subgroups are reported in Supplementary Table S2.

Fig. 1 shows the effects of these novel treatment patterns on the observed relative survival among males 65–74 (the largest patient group, who are also most likely to be screening-eligible) for the 2012–2017 and 2018–2023 periods. Stage IV Adenocarcinoma 3-year relative survival more than doubled from 6.5 to 14.1%. Patients with stage II–III cancers also see increases in survival ranging from 2 to 13 percentage points. Survival benefits are lowest among small cell lung cancer cases, for whom no novel therapies were approved in the observation period. Supplementary Table S2 presents relative survival for other patient-groups. Although survival is broadly improved in the 2018–2021 period, prognosis for stage IV lung cancer remains poor, with only 6–14% survival after 3 years by patient group for squamous cell carcinoma, and 8–26% for Adenocarcinoma.

**Fig. 2:**
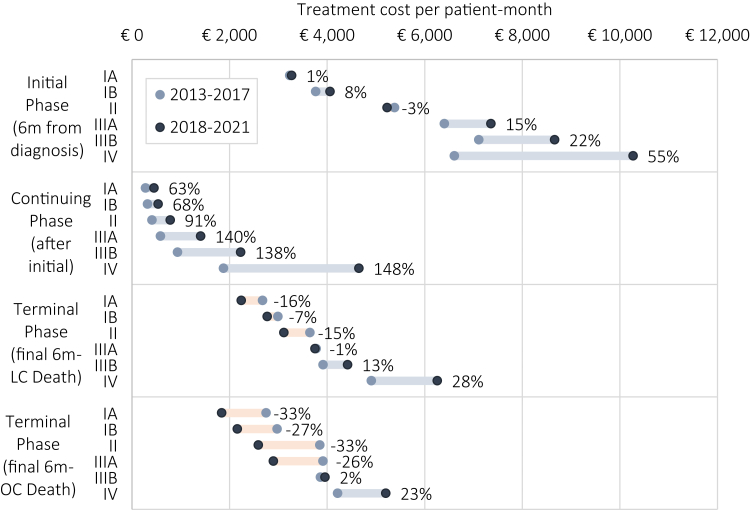
**Treatment cost per patient-month by phase of care and stage of cancer at diagnosis, and relative increase by period.** Figure 2 shows the increase in patient-month expenditures by phase of care in the 2018–2021 period, relative to the 2013–2017 period (note that whereas the survival model includes person years in 2012 and 2022–2023, cost data were available 2013–2021). Estimates are obtained using regression modeling of medical expenditures of individual-level claims data using a nationwide (n = 19.2 m) dataset, linked to cancer registry data of lung cancer incidence and tumor characteristics (n = 137,129). The terminal care (LC) phase represents the patient-month cost of the final 6 months of life for patients recorded to die of lung cancer, for the (OC) cases it represents patient-month cost for those reported to die of other causes. The initial phase represents the first 6 months of treatment. The continuing phase represents any intermittent care between the initial and terminal phase, up to 5 years. Exact methods, as well as estimates by histology, sex and treatment regimen are reported in our previous publication.^7^ Cost values here are shown in their original 2021 price index level. For application to the expected cost of treatment in MISCAN simulations, values are scaled to the 2023 price index level.

In the same period, lung cancer treatment expenditures were found to increase by 52% on average per patient,^7^ as shown in Fig. 2. Particularly treatment in the initial care phase of stage IV cancers increased, namely by 55% per patient-month. The continuing phase of stage IV cancers, between initial and terminal treatment, is 148% more expensive in the 2018–2021 period, demonstrating the prolonged nature of targeted- and immunotherapy treatment relative to previous treatment practice. Whereas previous treatment regimens concentrate expenditures to a greater extent in the initial or terminal phase, novel treatment regimens extend expensive treatment into the continuing care phase.^39^ Stage I–II treatment expenditures were increased mostly in the continuing phase (63–91%), which may correspond to novel therapy use after progression post-surgery. A complete accounting of changes in expenditures per patient-month is reported in Supplementary Table S3.

### The cost-effectiveness of lung cancer screening with novel treatment patterns

The MISCAN-Lung model simulated lung cancer outcomes for 5 scenarios: no screening, annual per TLHC eligibility criteria, biennial per TLHC criteria, annual per USPSTF criteria and biennial per USPSTF criteria. Table 1 reports summary outcomes of the benefits and costs relative to no screening of each screening scenario. Each strategy studied would be cost-effective relative to no screening at a willingness to pay of €20,000 per QALY. Scaled to the size of Dutch cohorts 1949–1979, the net monetary benefit of a A-TLHC screening program would be 2.21 billion.

**Table 1:** Incremental benefits and cost of exemplary screening strategies relative to no screening, per 100,000 population.

	Screening strategy and treatment model							
	A-TLHC		B-TLHC		A-USPSTF		B-USPSTF	
	Old treat-ment	Novel treat-ment	Old treat-ment	Novel treat-ment	Old treat-ment	Novel treat-ment	Old treat-ment	Novel treat-ment
Benefits of screening								
LC deaths prevented (% of total)	569 (10.8%)	563	408 (7.7%)	403	838 (15.8%)	826	601 (11.3%)	592
		−1.1%		−1.1%		−1.5%		−1.5%
LY gained (discounted)	4276	4118	3025	2912	5709	5494	4070	3916
		−3.7%		−3.7%		−3.8%		−3.8%
QALY gained (discounted)	3362	3253	2353	2276	4469	4321	3149	3044
		−3.2%		−3.3%		−3.3%		−3.3%
Net cost (€) relative to no screening (discounted)								
CT screening	23.4 m	23.4 m	12.3 m	12.3 m	45.2 m	45.2 m	23.6 m	23.6 m
Follow-up	0.59 m	0.59 m	0.59 m	0.59 m	0.72 m	0.72 m	0.72 m	0.72 m
Risk-assessment	0.07 m	0.07 m	0.07 m	0.07 m	0.08 m	0.08 m	0.08 m	0.08 m
Care for stage I–II cancers	30.1 m	34.3 m	21.6 m	24.8 m	44.0 m	49.4 m	31.6 m	36.1 m
Care for stage III–IV cancers	−6.6 m	−18.7 m	−1.8 m	−8.7 m	−7.7 m	−23.3 m	−1.2 m	−9.8 m
Total	47.6 m	39.7 m	32.7 m	29.1 m	82.3 m	72.2 m	54.9 m	50.7 m
		−16.7%		−11.0%		−12.3%		−7.6%
Cost (€) per LY gained	11,142	9639	10,810	9996	14,419	13,135	13,477	12,938
		−13.5%		−7.5%		−8.9%		−4.0%
Cost (€) per QALY gained	14,174	12,202	13,900	12,791	18,422	16,701	17,422	16,644
		−13.9%		−8.0%		−9.3%		−4.4%

Table 1 reports summary outcomes of four exemplary screening strategies: annual (A-TLHC) and biennial (B-TLHC) screening per TLHC (Targeted Lung Health Check) eligibility criteria (ages 55–75 from 1.51% PLCOm risk), and annual (A-USPSTF) and biennial (B-USPSTF) screening per USPSTF (United States Preventive Services Task Force) eligibility criteria (ages 50–80 from 20 packyears and no more than 15 y since smoking cessation). Results represent screening implementation from 2023 onwards for Dutch birth cohorts 1945–1979. Lifetime cost of screening and treatment are discounted by 3% to present value in 2023, and scaled per 100,000 individuals alive at the start of screening. For each strategy, the cost and Quality Adjusted Life Years Gained (QALYG) are given using survival and treatment costs from before the introduction of novel lung cancer therapies (2012–2017), and after updating both survival and cost to post 2018 values. Categorized costs are derived from unit costs presented in Supplementary Table S1.

Relative to no screening, the primary costs incurred by screening implementation are treatment for stage I–II cancers, as well as CT screening cost. Treatment cost for stage III–IV are reduced in the screening scenarios, as fewer cancers are detected in an advanced stage. For A-TLHC, novel treatment patterns reduce the LY gained due to screening by 3.7%, and QALY gained by 3.2%. The expected number of lung cancer deaths prevented decreased 1.1%. Contemporary survival patterns reduce the relative benefit of early detection, as life expectancy for lung cancer patients in the no screening scenario lengthens. Concomitantly, the unit cost of a person month of late-stage cancer is increased, as the treatment regimen is expected to be maintained for longer. As a consequence, the expected cost savings of the A-TLHC scenario from preventing late-stage treatment increased from 6.6 million/100,000 population under previous treatment, to 18.7 million.

**Fig. 3:**
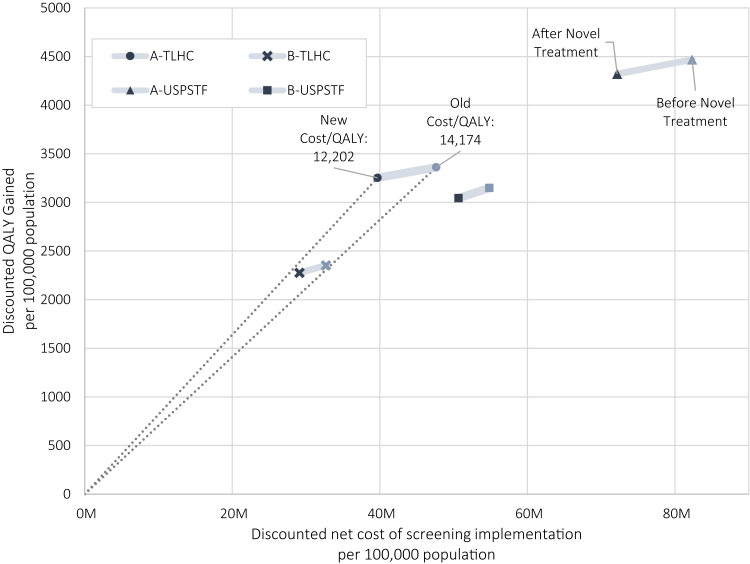
**Novel therapies decrease cost of screening more than QALY gained.** Figure 3 presents the discounted Quality Adjusted Life Years (QALY) gained relative to no screening, as well as the discounted cost of four exemplary screening strategies: annual (A-TLHC) and biennial (B-TLHC) screening per TLHC eligibility criteria (ages 55–75 from 1.51% PLCOm risk), and annual (A-USPSTF) and biennial (B-USPSTF) screening per USPSTF eligibility criteria (ages 50–80 from 20 packyears and no more than 15 y since smoking cessation). Results represent screening implementation from 2023 onwards for Dutch birth cohorts 1945–1979. Lifetime cost of screening and treatment are discounted to present value in 2023, and scaled per 100,000 individuals alive at the start of screening. For each strategy, the cost and QALYG are given using survival and treatment costs from before the introduction of novel lung cancer therapies (2012–2017), and after updating both survival and cost to post 2018 values.

Fig. 3 demonstrates the change in cost-effectiveness of lung cancer screening on the cost-QALY plane for each strategy. We found that the effect of novel treatment patterns bears stronger on the expected cost than on the expected QALY gained. This is most pronounced for the annual screening strategies. For A-TLHC, the expected cost per QALY gained reduced from 14,172 to 12,201, a 13.9% difference. A-USPSTF includes a larger screening population and is therefore slightly less cost-efficient at 18,421 per QALY, reducing to 16,701 after incorporating novel treatment patterns.

### Sensitivity analyses

We test the sensitivity of our primary outcome, the cost-effectiveness of the A-TLHC strategy, by varying key input parameters. Incremental cost and benefits of CT screening are presented in Table 2. We find the cost-effectiveness particularly sensitive to the discount rate, consistent with the long time horizon of particularly the benefits of population screening. Using differential discounting as recommended by the Dutch Health Care Institute^40^ (i.e., discounting benefits at a lower 1.5% rate), we find A-TLHC to cost only 9002 per QALY. Conversely, a higher discount rate of 6% increases the cost per QALY 52%.

**Table 2:** Incremental benefits and cost of annual screening with Targeted Lung Health Check eligibility criteria under various sensitivity analyses of model parameters.

	A-TLHC benefits and cost						
	QALY	LY	Cost (€)	Cost/QALY (€)	Diff. with baseline (%)	Cost/LY (€)	Diff. with baseline (%)
Before novel treatment	3362	4276	47.6M	14,174	16.2	11,142	15.6
Baseline (after novel treatment)	3253	4118	39.7M	12,202	0.0	9639	0.0
Updating survival only	3253	4118	43.4M	13,339	9.3	10,537	9.3
Updating cost only	3362	4276	48.4M	14,410	18.1	11,328	17.5
Discount rate–0%	6106	7596	47.2M	7732	−36.6	6215	−35.5
Discount rate–6%	1875	2427	34.9M	18,592	52.4	14,360	49.0
Discount rate–3% for cost, 1.5% for benefits^a^	4409	5530	39.7M	9002	−26.2	7178	−25.5
Stage IV terminal cost applied to stage I–II^b^	3253	4118	40.2M	12,350	1.2	9756	1.2
Stage I–II treatment cost +30%	3253	4118	50.0M	15,362	25.9	12,135	25.9
Stage I–II treatment cost −30%	3253	4118	29.4M	9042	−25.9	7143	−25.9
Stage III–IV treatment cost +30%	3253	4118	34.2M	10,517	−13.8	8308	−13.8
Stage III–IV treatment cost −30%	3253	4118	45.2M	13,886	13.8	10,969	13.8
CT and administration cost +30%	3253	4118	46.7M	14,355	17.6	11,340	17.6
CT and administration cost −30%	3253	4118	32.7M	10,049	−17.6	7938	−17.6
Attendance rate 50%^c^	1839	2339	23.0M	12,526	2.7	9848	2.2
VAS utility^d^	3110	4118	39.7M	12,763	4.6	9639	0.0

Table 2 reports the Quality Adjusted Life Year (QALY) and LY of annual screening with Targeted Lung Health Check (A-TLHC) eligibility criteria (ages 55–75 from 1.51% PLCOm risk), relative to no screening. The baseline result is reported, as well as sensitivity analyses pertaining key model inputs such as the treatment cost, the discount rate of benefits and harms, the assumed attendance at lung cancer screening events, and the norm utility instrument used.

a Differential discounting of benefits and harms is recommended by the Dutch National Health Care Institute.^40^

b Applies the cost of terminal-phase lung cancer care from stage IV of the same histology to all terminal-phase care for stage IA–II lung cancer.^40^

c In a reduced-attendance scenario, we assign half of no-shows to structural non-attenders, and distribute the other half stochastically to the remaining screening population.

d Use of Visual-Analog-Scale-derived health utilities for the population-averages by age and sex group, rather than the EQ-5D baseline.^30^

Treatment patterns, and their associated expenditures per patient month, may continue to develop as new therapies are introduced and existing therapies are extended to a larger patient population. If all cost of treatment for stage I–II cancer increase 30%, the screening would become less cost-efficient, with a 25.9% higher ICER. A lower attendance rate of 50% is only expected to reduce cost-efficiency by 2%; although gross QALY gained would decrease 43.5%, the primary cost drivers of CT screening and early-stage treatment would decrease in par. Across all additional analyses, we find no scenario that would cause screening to exceed a common willingness-to-pay threshold for secondary prevention of €20,000.

## Discussion

Here, we performed a cost-effectiveness analysis of lung cancer screening in the Netherlands, considering the effect of novel treatment patterns on the incremental cost and benefit of screening. We expand on the current health economic literature on lung cancer screening, which finds lung cancer screening to be cost-effective overall, but has not yet used empirical data for the cost and survival impact of novel therapies.^13^, ^14^, ^15^^,^^18^^,^^41^ We also add to recent modeling evidence that incorporated trial-based estimates of the survival benefit as well as unit costs of treatment,^16^^,^^42^, ^43^, ^44^, ^45^ by employing empirical treatment costs derived from the entire Dutch lung cancer patient population.^7^

We find novel treatment patterns for lung cancer to have two opposing effects on the cost-to-benefit ratio of CT screening. Expected benefits in LY or QALY gained are expected to decrease 3.2–3.8%, but expected costs are expected to decrease even further, by 7.6–16.7%. For our estimates of the cost-effectiveness of annual screening, but with UK Targeted Lung Health Check eligibility (screening ages 55–75 for those with >1.51% PLCOm risk), this resulted in a cost per QALY relative to no screening that was 13.9% lower than before the introduction of targeted- and immunotherapies. Biennial screening, as maintained in the UK TLHC,^23^ was found to be less cost-efficient than annual screening. However, we do not yet account in the MISCAN-lung model for the potential for personalizing the screening interval, which may prove an efficient middle ground between annual and biennial screening. Results from the ongoing 4-IN-THE-LUNG-RUN trial may inform whether a biennial personalized program may be as effective as annual screening.^46^

The observed survival data for the cohort exposed to novel therapies are encouraging. At 3 years from diagnosis, some patient groups have over double the survival seen before. However, when considering absolute over relative improvements, these results also offer a sobering view of stage IV survival, which stands below 30% for each patient subgroup included in our analysis. For these individuals, particularly those aged over 75 who are commonly offered only best supportive care, early detection of the disease may offer more opportunity for prolonging life than current treatment innovations. Concurrently, treatment expenditures are increasing rapidly, and new therapies are continually under development and reimbursement consideration. For example, immunotherapy is increasing administered as adjuvant therapy for stage II–III lung cancer.^47^^,^^48^ Future research should continue to audit the real-world cost and survival of lung cancer to test the efficacy of reimbursed care, and to incorporate these estimates in evaluating the financial prudency of early detection or prevention strategies. It should also be considered in evaluating screening modalities whether the treatment landscape in which the modality was evaluated is representative of the target context or period of implementation. However, we find the impact of recent improvements in treatment effectiveness on the lung cancer deaths prevented and (quality-adjusted) life-years gained to be modest. Furthermore, the high costs associated with these novel therapies considerably improve the cost-effectiveness of lung cancer screening.

Our study had certain limitations in scope and methodology. The treatment expenditures used reflect unit prices as reimbursed by Dutch insurance. Collective price agreements, the benefits of which are distributed lump-sum at the end of each year are therefore not included. These pricing agreements for specific medicines are obscured to the public. Although these may reduce the cost impact of novel therapies, lung cancer care expenditures are increasing at a greater rate than the total lump sum discount for the Dutch health sector.^7^ We also do not have data to stratify the cost by the profile of the lung cancer patients, which may leave differences undetected in treatment and costing patterns between those eligible and ineligible for screening. Additionally, treatment patterns and screening efficacy data by socioeconomic status could help identify the health equity effects of screening relative to current treatment practice, which is widening socioeconomic disparities.^49^^,^^50^ Our survival models make use of relative survival of lung cancer patients, calculated by subtracting from the overall survival the cohort-specific expected survival. Smoking-related comorbidities may influence the accuracy of this method, requiring specialized life tables for health-behavioral peers of lung cancer patients. Additionally, the socioeconomic composition of a lung cancer patient cohort could warrant further stratification of the reference life tables. Although this was previously found insignificant due to the short overall survival in lung cancer,^51^ future research should revisit this topic under novel treatment regimens. Our study also considered a limited scope of four potential screening strategies. Additional analyses may investigate a broader range of strategies to determine the optimal strategy of screening before and after novel treatment patterns. Since we found the treatment landscape to have a strong bearing on the cost per QALY, it may also influence the optimal frequency, starting and stopping ages of screening.

To conclude, novel therapies in lung cancer affect both the benefits and cost of a potential CT screening program in the Netherlands. We find the quickly increasing treatment expenditures outpace any potential loss in screening benefit due to the lower number of LY gained. Currently, metastatic lung cancer is still a leading public health problem that is becoming increasingly expensive to treat. CT screening for lung cancer provides a cost-effective method of advancing lung cancer diagnosis to a stage with better patient outcomes, and reduced medical cost.

## Contributors

All authors contributed to conceptualization and writing - review and editing. Kevin ten Haaf, Carlijn van der Aalst, and Harry de Koning contributed to project administration and funding acquisition. Kevin ten Haaf contributed to supervision and methodology. Koen de Nijs performed investigation, methodology, validation, visualization, and writing of the original draft. Kevin ten Haaf and Koen de Nijs verified the underlying data and statistical analysis. All authors read and approved the final version of the manuscript.

## Data sharing statement

Results are based on calculations by researchers of the Erasmus Medical Center using non-public microdata from Statistics Netherlands. The microdata can be requested through an application to Statistics Netherlands and with the Netherlands Cancer Registry. MISCAN model outputs can be made available upon reasonable request.

## Declaration of interests

Koen de Nijs reports grants from the NIH, the University of Zurich and the European Commission. Dr. Kevin ten Haaf reports grants from the NIH, the University of Zurich, Cancer Research UK, Cancer Australia, the Australian Ministry of Health, presentation fees from the Centr Hospitalier Universitaire Vadois, Johnson & Johnson and the Deutsches Krebsforschungszentrum, as well as travel support from the rescue lung society, and the international association for the study of lung cancer. Dana Moldovanu reports no disclosures. Juul Hubert reports no disclosures. Isabelle van den Bosch reports no disclosures. Anouk Eijkelboom reports consulting fees from AMGEN. Carlijn van der Aalst reports grants from the NIH, speaking fees from KALCIO Healthcare and Longkankernet, travel support from WHO-IARC and the LUKA project, and advisory board membership to B3Care and SOLACE. Harry de Koning reports grants from the NIH, speaker fees from AstraZeneca, advisory board membership to SOLACE.

## Appendix A Supplementary data

Supplemental Materials

LC_Treatment_Manuscript_clean_LBK (Page no: 22–26)
