# Supplementary material for: Influence of changing patterns in lung cancer treatment and survival on the cost-effectiveness of CT screening: a modeling study
Source: eClinicalMedicine. 2025 Aug 29;88:103446. doi: 10.1016/j.eclinm.2025.103446 (PMC12418876; doi:10.1016/j.eclinm.2025.103446)
Supplement: Supplemental Materials [file mmc1.docx]

# Supplement to “*Do changing patterns in lung cancer treatment influence the cost-effectiveness of ct screening*?”

Koen de Nijs^1^, Kevin ten Haaf^1^, Dana Moldovanu^1^, Juul Hubert^1^, Isabelle van den Bosch^1^, Anouk Eijkelboom^2^, Carlijn van der Aalst^1^, Harry J. de Koning^1^

1: Department of Public Health, Erasmus MC - University Medical Center Rotterdam, 3015 CE Rotterdam, the Netherlands

2: Department of Research and Development, Netherlands Comprehensive Cancer Organisation, Rijnkade 5, 3511 CV Utrecht, the Netherlands.

Inhoud

[Supplement to “*Do changing patterns in lung cancer treatment influence the cost-effectiveness of ct screening*?” 1](#_Toc190874337)

[Methodological Supplement 2](#_Toc190874338)

[Model summary and structure 2](#_Toc190874339)

[Smoking history generator 3](#_Toc190874340)

[Death from other causes 3](#_Toc190874341)

[Smoking dose-response relationship for lung carcinogenesis 4](#_Toc190874342)

[Natural History of Lung Cancer 4](#_Toc190874343)

[Relative Survival of Lung Cancer by Stage, Histology and Period of Diagnosis 6](#_Toc190874344)

[Computed Tomography Screening 6](#_Toc190874345)

[Integrating Modules 7](#_Toc190874346)

[Model Outcomes 11](#_Toc190874347)

[Treatment cost before and after the introduction of novel therapies 11](#_Toc190874348)

[Dutch smoking behavior and lung cancer epidemiology 15](#_Toc190874349)

[NELSON Screening Effectiveness 18](#_Toc190874350)

[References 19](#_Toc190874351)

# Methodological Supplement

## Model summary and structure

To estimate the effectiveness of lung cancer screening before and after the introduction of novel lung cancer therapies, we used the MIcrosimulation SCreening ANalysis (MISCAN) Lung model, a stochastic, microsimulation model. In brief, the model simulates individual life histories in the considered population from birth until death, in the presence or absence of a screening program. Through comparing the life histories in the presence of screening with the corresponding life histories in the absence of screening, MISCAN-Lung can estimate the effectiveness and costs of screening scenarios. MISCAN-Lung was calibrated to individual-level data from the National Lung Screening Trial (NLST) and the Prostate, Lung, Colorectal, and Ovarian Cancer Screening Trial (PLCO),^1^ ^2^ and has since been recalibrated to the Nederlands-Leuven Longkanker Screening Onderzoek (NELSON).^3^ Estimates of the preclinical sojourn time by stage, and the probability of lung cancer detection are now matched to outcomes from the NELSON trial, per methods elaborated on in a future section. The model is programmed in Python 3.

MISCAN-Lung is a semi-Markov model, which generates durations for each state. Individuals are simulated one at a time, which allows future state transitions to depend on past transitions giving the model a “memory”. MISCAN-Lung simulates sequences of events by drawing from distributions of probabilities/durations, which makes the results of the model subject to random variation. The model is calibrated such that the aggregate of all these individual outcomes matches the smoking behavior patterns and lung cancer incidence across different sexes and birth cohorts of the Netherlands, and the outcomes of the PLCO, NLST and NELSON trials. For smoking behavior prevalences, we use the Dutch Health Survey for years 1989 to 2020 to establish proportions of current, former and never smokers over time for each 5-year birth cohort and sex separately.^4^ Lung cancer incidence is taken from the Netherlands Cancer Registry, covering all incident lung cancers 2000-2020 in the Netherlands by stage and histology of cancer.^5^

MISCAN-Lung consists of several modules: a demography/smoking history generator module, a smoking-dose response module for lung carcinogenesis, a natural history module and a screening module. The workings of each of these modules will be discussed in detail in the coming sections. Afterwards, we discuss how they were adjusted to Dutch cohorts 1942-1961.

## Smoking history generator

First, birth-tables, representative for the population under consideration, are used to draw a date of birth for each simulated individual. Age, sex and five-year birth-cohort specific smoking initiation probabilities, representative for the population under consideration, are used to determine whether an individual initiates smoking and the age of smoking initiation. Upon smoking initiation, persons enter one of five increasing smoking intensity categories. Age, sex, five-year birth-cohort and smoking intensity category specific by averaged number of cigarettes smoked per day are generated for each individual that initiates smoking. If an individual initiates smoking, age, sex and cohort specific smoking cessation probabilities are used to determine whether an individual ceases smoking and the age of smoking cessation.

## Death from other causes

Upon generating a person’s smoking history, the age of death from causes other than lung cancer is generated, using mortality probabilities based on the person’s smoking history (smoking duration, smoking intensity category and average number of cigarettes per day, smoking status and years since cessation, if applicable), year of birth, age and sex. For example, for the Male 1960 cohort, Figure 1 shows the mortality rate for never-smokers relative to current smokers for each smoking intensity quintile. After smoking cessation, individuals are assigned a mortality probability in between the never- and current-smoekr mortality probability, weighted by their years since smoking cessation. The maximum age an individual can achieve in MISCAN-Lung is exactly 100 years.

Figure 1 - Other-Cause Mortality Probability by Age and Smoking Status for the Male 1960 cohort

## Smoking dose-response relationship for lung carcinogenesis

The smoking-dose response module allows modeling lung carcinogenesis as a function of a person’s age, gender and smoking history. MISCAN-lung utilizes the two-stage clonal expansion model (TSCE) as described by Heidenreich et al., as its smoking-dose response module (which estimates a person's risk of lung cancer, as a function of age and smoking history).^6^ The parameters of the TSCE were obtained through calibration to the Nurses’ Health Study and the Health Professionals Follow-up Study ^7^, and were further calibrated to Dutch lung cancer incidence, smoking behavior, and individual-level outcomes from the NELSON study. ^4, 5^

## Natural History of Lung Cancer

Lung cancers are assumed to progress sequentially through stages IA to IV, as shown in Figure 2. The probability that a lung cancer progresses to a more advanced preclinical stage or is diagnosed clinically (e.g., diagnosed due to symptoms) is modelled by histology and stage. After clinical diagnosis, lung cancer survival is simulated using sex-, stage-, and histology specific survival estimates, obtained from the Netherlands Cancer Registry, per methods specified in the next section.^5^ The date of death for individuals with lung cancer is set to the earliest simulated date of death (either due to lung cancer or other causes).


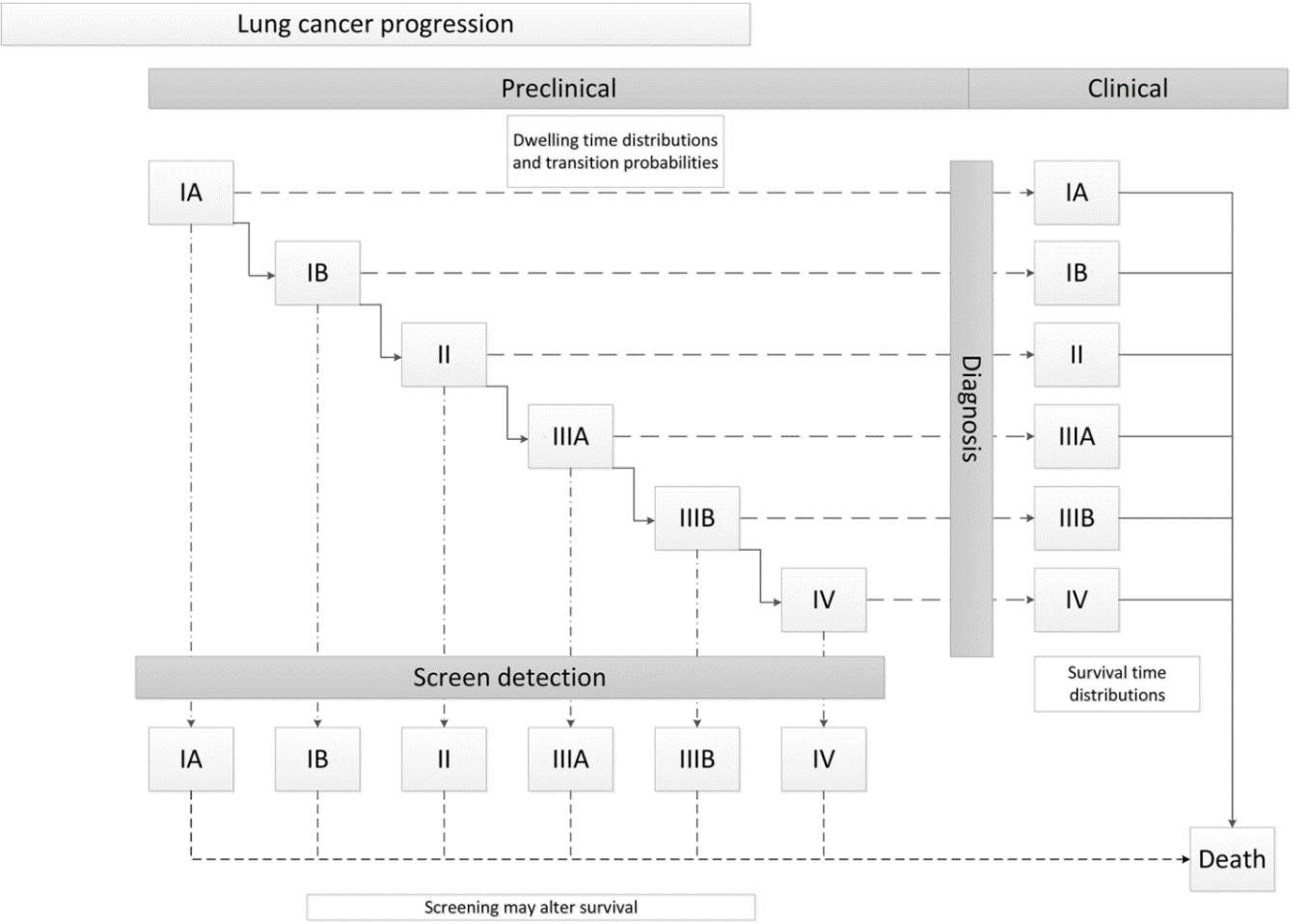


Figure 2 Lung cancer progression in the MISCAN-Lung model

Figure notes: Once lung cancer has developed, it will progress from less advanced to more advanced preclinical stages until it is clinically detected. This process is similar for all histologies, however, the average time spent in the current state differs by histology, preclinical cancer stage and gender. The probability that a cancer progresses to a more advanced preclinical stage or is diagnosed clinically (e.g., diagnosed due to symptoms) is modelled by histology and stage. Screening may detect cancers in each of the preclinical screen-detectable states, depending on the sensitivity of the screening test for the specific histology and preclinical detectable state. Upon detection of lung cancer by screening, a person’s life history may be altered. Detection by screening may prevent the lung cancer death, allowing them to resume their normal (lung cancer free) life history. The probability of lung cancer mortality prevention differs by the stage of detection. After clinical detection the patient’s duration of survival follows a histology and stage specific survival function, which is piecewise uniformly distributed. A screen detected case without successful mortality prevention is assigned the same age of lung cancer death from the life history without screening (in which the cancer is detected clinically at a later age). A person may also die from causes other than lung cancer.

## Relative Survival of Lung Cancer by Stage, Histology and Period of Diagnosis

For this particular analysis, which focuses on the consequences of novel treatment patterns on lung cancer screening, we used period-specific relative survival curves. As specified in the main text: incident lung cancer cases from 2012-2021 are included, with follow-up of survival until the 1^st^ of February 2023. The monthly relative survival is calculated using the Ederer-II method^8, 9^, adjusting the observed survival to the contemporary expected survival reported by Statistics Netherlands.^10^ Survival estimates are stratified by age at diagnosis (<65, 65-75, 75+), biological sex, stage of cancer (IA, IB, II, IIIA, IIIB, IV) and histology (Adenocarcinoma, Squamous Cell Lung Cancer, Other NSCLC, Small Cell Lung Cancer). Results are generated by period, with separate estimates for patient-years in the 2012-2017 period, and patient-years lived in the 2018-2023 period.

To smooth the relative survival data, a generalized linear model is fitted to each subgroup-specific curve, assuming a Poisson survival rate. The resulting period-specific survival curves are applied to the life histories of individuals with lung cancer generated in the MISCAN model to generate estimates of the lung cancer mortality rates in a screening and no-screening scenario, before and after the introduction of novel therapies.

For the 2012-2017 period, 72872 individuals are included with 63341 observed deaths. For the 2018-2021 period (noting that follow-up was available until 01-02-2023), a total of 74033 individuals are included with 47351 deaths. There was ample sample size to estimate age-, period-, stage- and histology-specific sensitivity overall. One exception was small-cell lung cancer in stage IA-II, which is very rare. For this cancer, the survival was grouped only by sex, period and histology, with stages IA-II assigned the same survival curve.

## Computed Tomography Screening

Screening may detect cancers in each of the preclinical screen-detectable states, depending on the sensitivity of the screening test for the specific histology and preclinical stage. The model parameters for CT sensitivity by preclinical stage and histology and the effectiveness of CT screening were calibrated to individual-level data from the NELSON trial.^3^ Upon detection of lung cancer by screening, a person’s life history may be altered. Detection by screening may prevent the lung cancer death, allowing them to resume their normal (lung cancer free) life history. The probability of successful mortality prevention differs by the stage at detection, and was also calibrated to individual-level outcomes from the NELSON study. Negative effects of screening, such as overdiagnosis of lung cancer (described subsequently), are also modelled.

## Integrating Modules

Figure 3 shows an example of how the model integrates the different modules to determine the benefits of screening. The demography/smoking history generator module first generates a date of birth, smoking history and date of death from causes other than lung cancer. This creates a life-history in the absence of lung cancer for Person 1 (shown in life history 1). The smoking-dose response module uses the simulated smoking history to determine whether and when lung carcinogenesis occurs for Person 1 (shown in life history 2). After lung carcinogenesis occurs, the natural history model generates the progression of the cancer, which is diagnosed because of symptoms in stage II in this example and results in a death due to lung cancer, before the death due to causes other than lung cancer would have occurred (shown in life history 1). In the screening module, a screening examination is simulated, as indicated by the arrow (shown in life history 3). The cancer is detected at the examination and, in this case, the earlier detection allows for successful treatment of the cancer. As a result, the lung cancer death is prevented, and the person’s life is prolonged.

Screening may also cause harms, as shown for Patient 2 in Figure 4. In Patient 2 lung cancer also develops, but the cancer would not have been clinically detected without screening (shown in life history 2). However, the cancer is screen-detected in stage IA during the screening examination simulated in the screening module (shown in life history 3). Thus, in this patient, screening detects a lung cancer that would have never become apparent during the patients’ lifetime if screening had not occurred, resulting in an overdiagnosed case. Thus, for Patient 2 screening does not provide any benefits, but results in life-years with lung cancer care that would not have occurred otherwise (overtreatment).


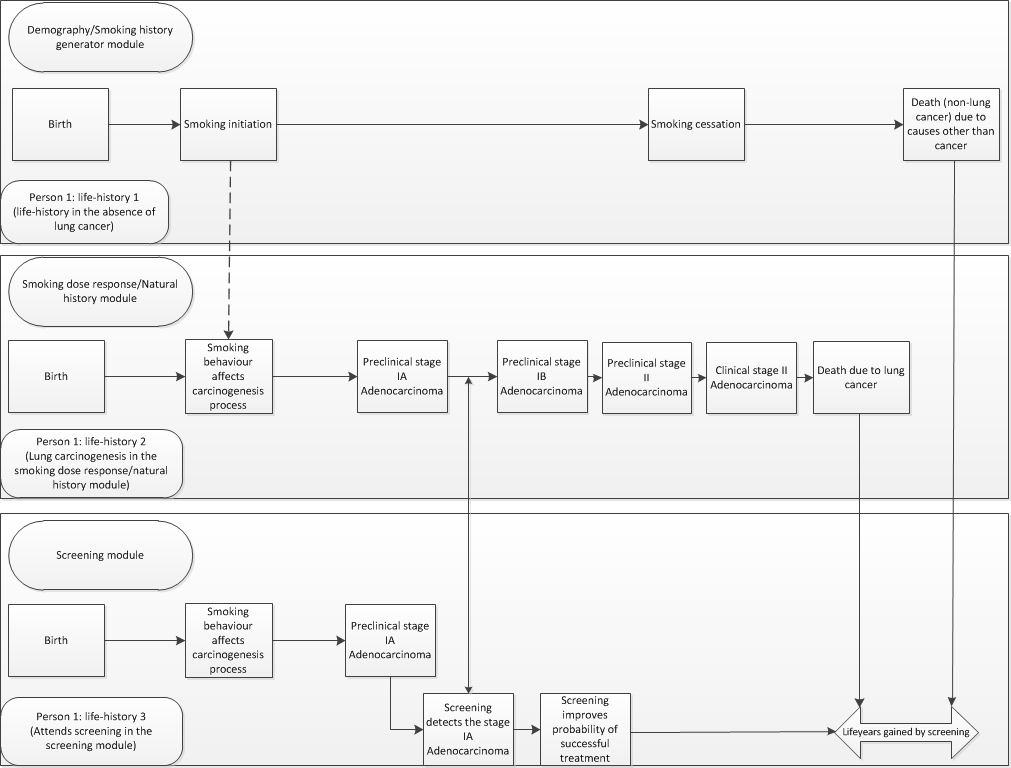


Figure 3 Integrating modules: modelling benefits of screening


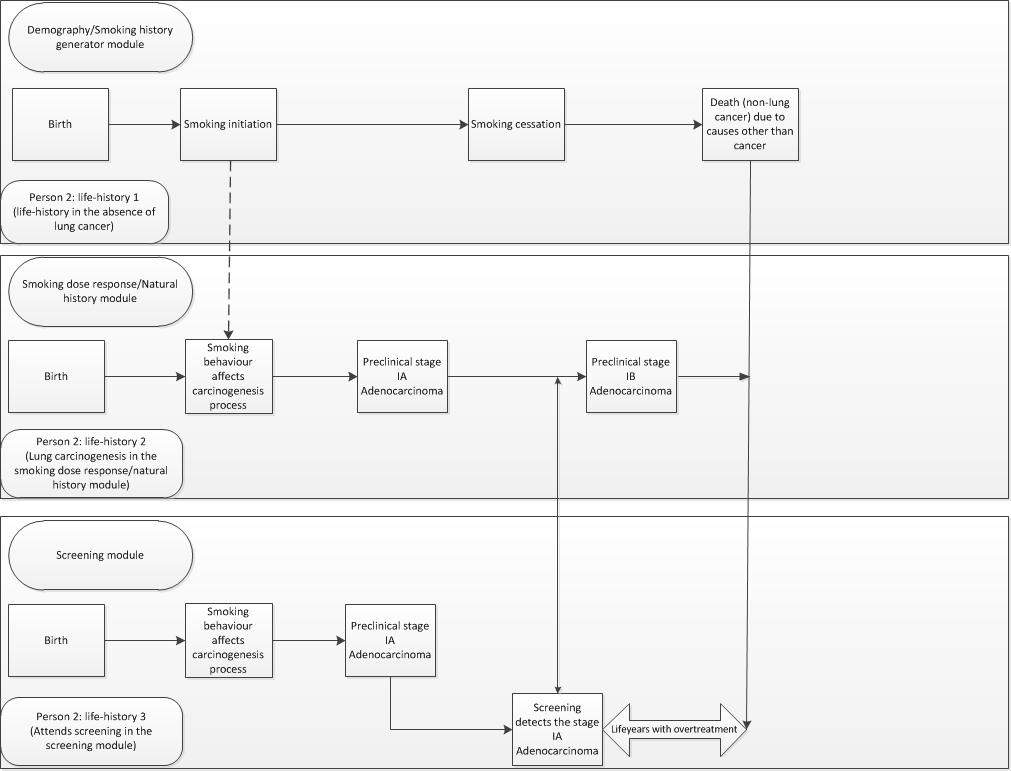


Figure 4 Integrating modules: modelling harms of screening

## Model Outcomes

The model outcomes presented in Table 1 of the main manuscript are generated as follows:

- **LC Deaths Averted:** The sum of lung cancer death events, less the sum of lung cancer deaths events in the scenario without intervention.
- **Life-years gained:** Remaining total life-years lived measured from the start of screening (2023), less the total life years in the no-screening scenario. Discounted by 3%.
- **Quality-Adjusted Life-years gained:** Remaining total life-years lived measured from the start of screening (2023), less the total life years in the no-screening scenario, adjusted for the health-related quality of life in the individual life course. Quality of life tariffs, reported in Supplementary Table 1, are used to adjust for the average quality of life at older age, as well as the decreased quality of life under lung cancer treatment. Discounted by 3%.
- **Net categorized cost relative to no screening:** The cost of screening relative to a no-screening scenario, from the start of screening onwards. Unit costs from Supplementary Table 1 are applied to screening events, follow-up exams, risk-assessment and lung cancer care by age-, period-, stage- and histology associated with the lung cancer case.

### Treatment cost before and after the introduction of novel therapies

We include particular focus on the treatment cost before and after the introduction of novel therapies in the Netherlands, as to include the monetary cost associated with the improved outcomes for particularly metastatic lung cancer. The complete methods underlying this costing study were reported previously.^11^ Summarized from the methodological supplement accompanying this paper, the methods are as follows.

#### Data (extended description)

Tumour-level information was supplied by the Netherlands Comprehensive Cancer Organisation (IKNL), who maintain the Netherlands Cancer Registry (NCR).^12^ The NCR has collected tumour-level information for cancer in the Netherlands since 1989. The data are gathered by data managers in each hospital in the Netherlands. All cancers minimally include information on the type of cancer, the stage of cancer and the first line of treatment. Information on further outcomes, such as recurrence or progression, is not commonly recorded, such that registration of the cancer with the NCR is finished within a year of incidence. We include all lung cancers (ICD-0 C33 and C34 codes), diagnosed 2012-2021 in the Netherlands for patients aged 40 and over. 137,129 tumours across 133,426 individuals were included. Tumour data include morphology, localisation, first-line treatment, as well as the TNM-stage per the staging manual contemporary to incidence. Cancer morphology was mapped to one of four broader histology categories of Adenocarcinoma, Squamous cell Carcinoma, Small-Cell Carcinoma and Other Non-Small Cell Carcinoma (morphology given in Supplementary Table 1). Treatment data includes a binary indicator variable of whether the individual received each of seven categories of care:

- No therapy – if this indicator is positive, all other indicators are negative.
- Surgery
- Radiotherapy
- Chemotherapy
- Targeted therapy – includes all immunotherapy
- Other systemic therapy
- Hormone therapy – collapsed in our analyses into the category `other therapy` together with `other systemic therapy` to adjust for the low sample receiving both categories of therapy.

More detailed treatment information, such as treatment complications and detailed information on the type of drug or surgery, is also supplied, but with imperfect coverage of our dataset.

Information on the tumour level was linked to individual-level data from Statistics Netherlands (CBS). Individual level information supplied by CBS include the following variables:

- Personal Records Database (BRP) birthyear, and sex.
- Date of death.
- Cause of death, available for any death from 2013. We include entries up to 31-12-2022, which gives the maximal follow-up for our determination of time spent in lung cancer care. We recode the information, which is specific to the World Health Organisation International Classification of Disease coding system, to all lung-cancer (“lung cancer”) and non-lung cancer death (“other causes”). Individuals with a date of death, but no registered cause of death (n=59) are assigned a third category of cause of death: “unknown”.
- Health expenditures, as described below.

Linkage of tumour-level information to CBS was excellent, with 3.8% (n=5,229) tumours not matched to a Statistics Netherlands record. Health care expenditures are collated and supplied to CBS by the health insurer collaborative VEKTIS. Annual expenditures on the individual level are recorded for 19 categories of health care provider. Expenditures include all health care reimbursed by the Dutch standard health insurance. Dutch standard health insurance includes an individual co-payment of minimally €385 annually. The individual co-payment is included in the sum reported by VEKTIS, such that the figure represents the total health care cost. Some health care in the Netherlands is not insured by the basic insurance, but through other avenues. There is separate insurance for long-term care. Additionally, some health care such as dental health care and physical therapy for non-chronic conditions is insured through supplementary insurance or paid for out-of-pocket.

Incidentally (0.01% of all expenditure entries), negative expenditure records occur, representing a retraction of an expense in an earlier year. In this case, the expense level was set to 0 for the relevant year, and the equivalent sum was subtracted from the preceding year. All expenditures are corrected for inflation to 2021 price levels.^13^ 77.6% of the total population (n=19.2m) are observed each year of 2013-2021. Most missing years occur at the start or end of the 2013-2021 window, likely representing a start or end to life in the Netherlands, with only 1.0% of individuals (0.3% among those with lung cancer) presenting a non-contiguous expenditure history. Of those with lung cancer, only 0.3% of individuals presenting a non-contiguous expenditure history. From the total expenditure dataset, we include all individuals with a lung cancer diagnosed in calendar years 2012-2020, as well as an additional 2.2% of the total population without lung cancer to serve as controls (n=387,085). To get a comparable control population, individuals are sampled with a probability proportional to the frequency of their birthyear and sex combination in the population with lung cancer, relative to the general population.

#### Methods

To determine the part of observed expenditures attributable to lung cancer, we model the increase in expenditures of individuals after incidence, relative to their age- and sex-adjusted expenditure levels of expenditures in years before lung cancer. After lung cancer incidence, we split up cancer care costs by phase of care (initial, continuing and terminal), per methods previously employed in the literature.^14, 15^ Individual expenditure levels before lung cancer are used as the offset, rather than controls from the population, to account for increased ex-ante expenditures associated with comorbidities common to lung cancer patients. To split the lung cancer attributable cost from annual individual expenditures, we use a random effects regression model, with annual expenditures as the dependent variable, and the months in that given year spent in lung cancer care as the independent variable. The regression may be represented by the formula

$$y_{i,t}=\alpha+x_{it}\beta+\upsilon_{i}+\varepsilon_{it}$$

where $y_{i,t}$ gives the total annual health expenditures of individual *i* at time *t*, $\alpha$ gives the intercept, $x_{it}$ contains the covariates (months spent in initial, continuing, terminal care (for other, unknown and lung cancer causes separately), indicator variables for the 5-year age and sex group of individual *i* at time *t*). $\upsilon_{i}$ reports the individual random effect to capture ex-ante individual variations in mean expenditures. $\varepsilon_{it}$ reports the error term, which we assume to be gamma distributed^[[1]](#footnote-1)^ to capture the typical distribution of expenditure variables. Additionally, the error are clustered to account for within-individual correlated errors. $\beta$ represents the vector of parameter estimates. We split up the months spent in initial, continuing and terminal care by different subgroups to study differences in the lung cancer attributable costs in $\beta$ by sex, stage of cancer, histology of cancer, period of incidence and type of first-line treatment.

The assignment of months of care to a calendar year allows us to account for the shift of treatment costs to the next calendar year when cancers are incident late in the year. Lung cancer care is split up into terminal care (final months of life, maximally 6), initial care (up to 6 months after incidence, not to coincide with terminal care) and continuing care (up to 5 years after initial care, not to coincide with terminal care). If survival is 6 months or less, only terminal care is assigned. If survival is 12 months or less, only terminal and initial care months are assigned. For survival over 12 months, any month more than 6 months from incidence and more than 6 months before death is considered a continuing care month, up to 5 years since initial care. Some costing studies have used longer periods of initial and terminal care. However, given that median survival for individuals observed to die within our observation window is only 7 months, we use 6 months for initial and terminal care as to prevent the terminal phase of care to constitute a dominating majority of all care months assigned to the individuals in our dataset. As a sensitivity analysis, we re-estimate excess expenditures with the maximal initial and terminal care phase length set to 12 months, presented in Supplementary Table 9 of the supplementary results.

The estimated association between the months spent in each phase of care represent the excess expenditures associated with one month of treatment, relative to pre-cancer cost levels, adjusted for the sex and age at the time of treatment.

We fit 5 cost models to our data, differing by the stratification of the estimated lung cancer attributable excess expenditures:

1. Excess expenditures by stage (IA, IB, II, IIIA, IIIB, IV) of cancer.
2. Excess expenditures by cancer stage and histology (Adenocarcinoma, Squamous-cell carcinoma, Small-cell Carcinoma and other Non-Small cell Carcinoma).
3. Excess expenditures by cancer stage, histology and sex (Male, Female).
4. Excess expenditures by cancer stage and period of treatment (2013-2017 relative to 2018-2021).
5. Excess expenditures by first-line treatment modality (Chemotherapy, Radiotherapy, Surgery, Targeted Therapy, Other and None).

To assign health expenditures to lung cancer care for a particular stage and histology of cancer, we need to select a primary cancer for individuals with multiple reported lung cancers. In total, 3,703 tumours occur among individuals with another lung cancer registered. Among those individuals, we are interested in determining the stage and histology of the cancer associated with the most intensive treatment, as well as any registered lung cancer death. To this end, we select as the primary cancer the cancer with the highest TNM stage, unless the cancer is incident more than 5 years before the later lung cancer. For example, a IB cancer is selected as the secondary cancer if a IA cancer is incident more than 5 years after. If a lung cancer death occurs, the terminal care months and associated expenditures are then assigned to the stage and histology of the later cancer. Alternatively, if a stage IIIB cancer is followed quickly by a stage II cancer, we attribute the expenditures to the stage and histology of the later-stage cancer. However, we use the incidence date of the earlier cancer to assign months spent in lung cancer care if the two cancers are within 12 months of each other. If the cancers are further apart, to prevent care for secondary cancers influencing the estimates of pre-cancer expenditure levels by age and sex, we include an indicator variable in $x_{it}$ which reports the number of months in the year spent after incidence of a secondary lung cancer.

## Dutch smoking behavior and lung cancer epidemiology

To calibrate the MISCAN-Lung model inputs to the Dutch setting, we evaluated the model’s potential to replicate Dutch lung cancer incidence. This ensures that model is representative of broader lung cancer epidemiology in the Netherlands.

We simulate lung cancer outcomes for the years 2000-2020 for cohorts 1935 to 1979. Smoking initiation, cessation and smoking-related other-cause mortality are calibrated to cohort life tables from the bureau of statistics and smoking prevalence per the Dutch Health Survey (1989-2020).^5, 16^ Microdata from the Dutch Health Survey informs our estimates of the quintiles of smoking intensity in cigarettes per day, by sex and 5-year cohort.^4^ National tobacco sales contemporary to the Dutch Health Survey are used to evaluate quantity underreporting by health survey respondents, yielding an estimate of 21% underreporting, consistent with previous estimates for other contexts^17-19^. Together, these estimates of smoking behavior are used to inform the Smoking History Generator component of the MISCAN-Lung model. The fit of the smoking history generator to observed current smoking prevalence over time by cohort are given in Figures 5 and 6.

For a given set of model inputs, 10,000,000 life histories are simulated. The sizes of the individual cohorts constituting this population are set to comply to 2010 cohort sizes per Statistics Netherlands. For each simulated individual, a smoking history and smoking related other-cause date of death are drawn. Depending on the smoking history, a lung cancer natural history may be established, using the Two-Stage Clonal Expansion model. Lung cancer outcomes are noted for the years 2000-2020 to compare to population-level calibration targets of lung cancer incidence. Simulated and recorded outcomes include lung cancer incidence and mortality by histology (adenocarcinoma, squamous cell carcinoma, other non-small cell lung cancer, and small cell lung cancer) and stage of cancer at incidence (stages IA, IB, II, IIIA, IIIB and IV). Figure 7 shows the lung cancer incidence generated after model calibration by the MISCAN-Lung model for the period 2000-2020, relative to observed values from the Dutch Cancer Registry.

**Figure 5 - MISCAN-Smoking History Generator (SHG) predictions of current smoking prevalence compared to observed Dutch Health Survey smoking prevalence for Male 10-year cohorts 1930-1970**


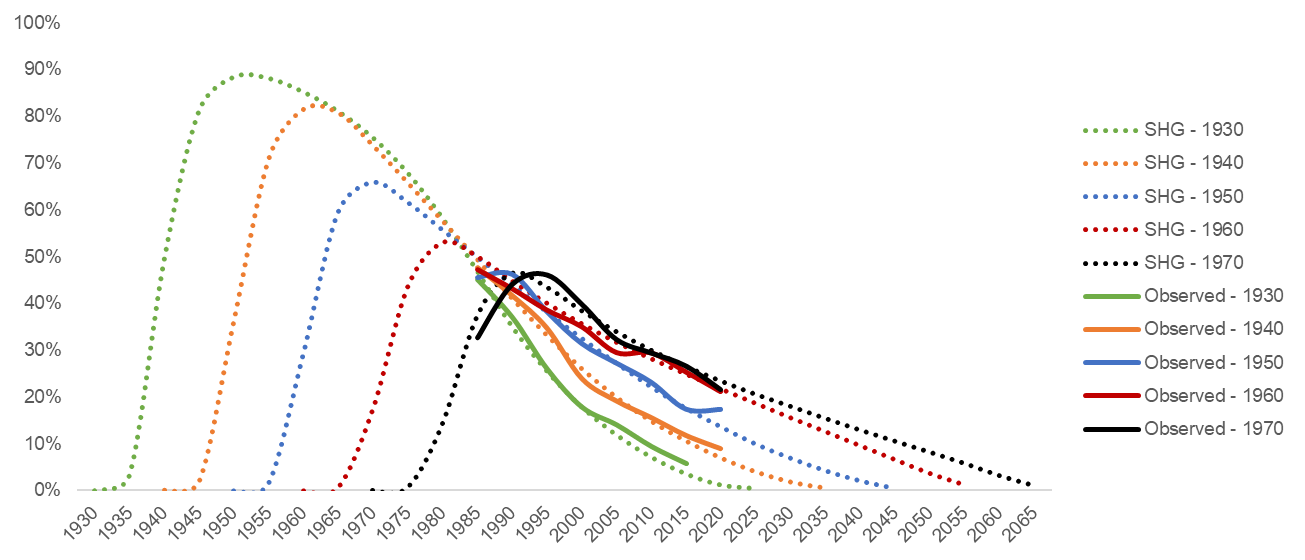


**Figure 6 - MISCAN-Smoking History Generator (SHG) predictions of current smoking prevalence compared to observed Dutch Health Survey smoking prevalence for Female 10-year cohorts 1930-1970**


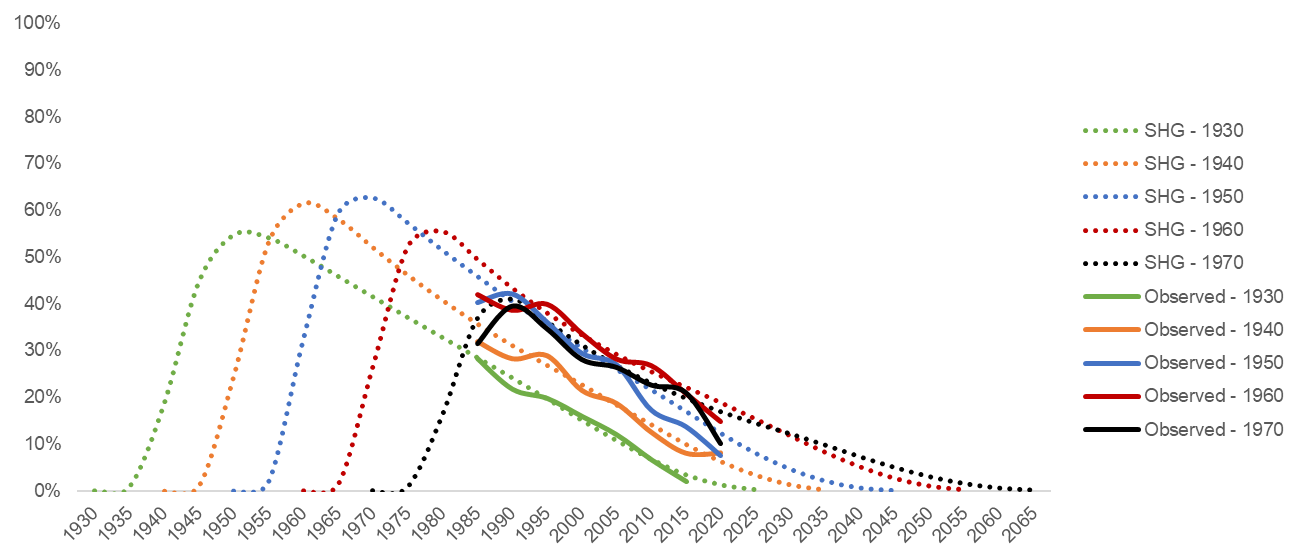


**Figure 7 - MISCAN-Lung simulated lung cancers by age and sex for the period 2000-2020 in the Netherlands.**


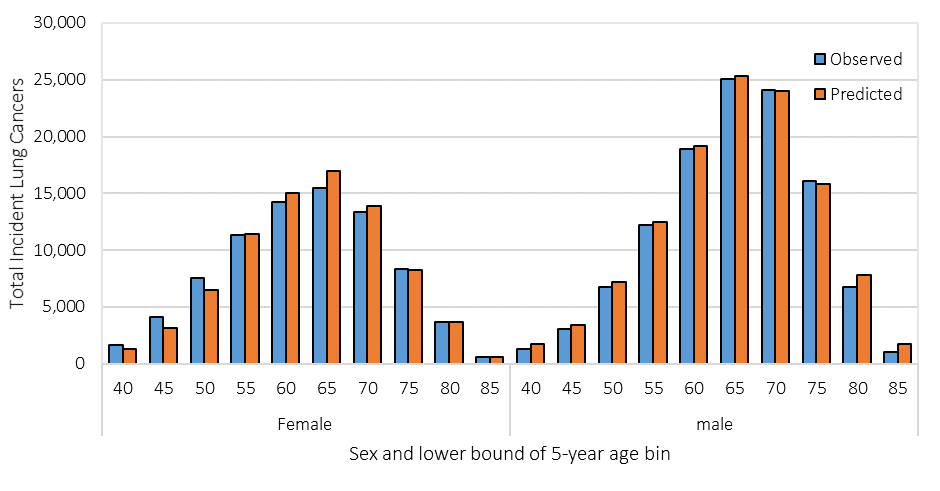


Figure 7 reports MISCAN-Lung predicted gross lung cancer incidence for the period 2000-2020 for the country of the Netherlands, summed across males and females. Dutch health survey data on smoking behaviour by sex for the 1930 to 1979 cohorts is used to inform the MISCAN-Lung microsimulation of life histories. Cohort sizes are based on the 2020 population composition of the Netherlands.

## NELSON Screening Effectiveness

Additionally, the model was calibrated to individual-level outcomes from the NELSON study to re-estimate parameters of screening effectiveness first calibrated to results from the NLST. ^1, 3^ The model calibration replicates the life history of each NELSON participant, and sets the MISCAN-Lung parameters of screening effectiveness to the values that best match the observed outcomes in the NELSON trial. Self-reported smoking histories were used to replicate lung cancer outcomes among simulated NELSON participant. Recalibrated parameters include the sensitivity of the CT screen by stage, as well as the preclinical sojourn time in stage IA Adenocarcinoma. NELSON calibration results suggest a higher sensitivity of CT screening for the detection of lung cancer than previous results from NSLT and PLCO trials, as well as a longer period of detectability in stage IA Adenocarcinoma. ^1, 2^ The complete NELSON-adjusted MISCAN-Lung parameter set and calibration methodology are being prepared for presentation in a forthcoming publication.^3^

## References

1. ten Haaf K, van Rosmalen J, de Koning HJ. Lung Cancer Detectability by Test, Histology, Stage, and Gender: Estimates from the NLST and the PLCO Trials. *Cancer Epidemiology Biomarkers &amp;amp; Prevention* 2015;**24**: 154.

2. Meza R, ten Haaf K, Kong CY, Erdogan A, Black WC, Tammemagi MC, Choi SE, Jeon J, Han SS, Munshi V, van Rosmalen J, Pinsky P, et al. Comparative analysis of 5 lung cancer natural history and screening models that reproduce outcomes of the NLST and PLCO trials. *Cancer* 2014;**120**: 1713-24.

3. de Nijs K, ten Haaf K, van der Aalst CM, Oudkerk M, de Koning HJ. OA05.04 A Comparison of Stage- and Histology-Specific CT Sensitivity in the NELSON Trial and the NLST. *Journal of Thoracic Oncology* 2022;**17**: S14.

4. Statistics Netherlands. Dutch Health Survey, 1989-2020.

5. Netherlands Cancer Registry (NCR). Lung Cancer Incidence and Survival. In: Netherlands Comprehensive Cancer Organisation (IKNL), ed., 2000-2012.

6. Heidenreich WF, Luebeck EG, Moolgavkar SH. Some properties of the hazard function of the two-mutation clonal expansion model. *Risk Anal* 1997;**17**: 391-9.

7. Meza R, Hazelton WD, Colditz GA, Moolgavkar SH. Analysis of lung cancer incidence in the nurses’ health and the health professionals’ follow-up studies using a multistage carcinogenesis model. *Cancer Causes & Control* 2008;**19**: 317-28.

8. Ederer F, Heise H. Instructions to IBM 650 programmers in processing survival computations: Methodological note, 1959.

9. Dickman PW, Coviello E. Estimating and modeling relative survival. *The Stata Journal* 2015;**15**: 186-215.

10. Statistics Netherlands. Mortality probabilities by age and sex, 2012-2023.

11. de Nijs K, de Koning HJ, van der Aalst C, Ten Haaf K. Medical costs of lung cancer by stage, histology and first-line treatment modality in the Netherlands (2012-2021). *Eur J Cancer* 2024;**208**: 114231.

12. Netherlands Comprehensive Cancer Organisation (IKNL). Netherlands Cancer Registry (NCR). In: (IKNL) NCCO, ed., 2012-2021.

13. Statistics Netherlands (CBS). Consumper prices; price index. In: Statistics Netherlands (CBS), ed. CBS Open data StatLine, 2013-2021.

14. Yabroff KR, Mariotto AB, Feuer E, Brown ML. Projections of the costs associated with colorectal cancer care in the United States, 2000-2020. *Health Econ* 2008;**17**: 947-59.

15. Sheehan DF, Criss SD, Chen Y, Eckel A, Palazzo L, Tramontano AC, Hur C, Cipriano LE, Kong CY. Lung cancer costs by treatment strategy and phase of care among patients enrolled in Medicare. *Cancer Med* 2019;**8**: 94-103.

16. Statistics Netherlands. Levensverwachting; geslacht, leeftijd (per jaar en periode van vijf jaren), 2020.

17. Pérez-Stable EJ, Marín BV, Marín G, Brody DJ, Benowitz NL. Apparent underreporting of cigarette consumption among Mexican American smokers. *American Journal of Public Health* 1990;**80**: 1057-61.

18. Gallus S, Tramacere I, Boffetta P, Fernandez E, Rossi S, Zuccaro P, Colombo P, La Vecchia C. Temporal changes of under-reporting of cigarette consumption in population-based studies. *Tobacco Control* 2011;**20**: 34-9.

19. Liber AC, Warner KE. Has Underreporting of Cigarette Consumption Changed Over Time? Estimates Derived From US National Health Surveillance Systems Between 1965 and 2015. *Am J Epidemiol* 2018;**187**: 113-9.

1. The gamma distribution accounts for the properties of cost values to have large positive outliers, but is only defined on the positive domain. As a consequence, we had trouble estimating the models for sparse health care expenditures, particularly the provider-specific results shown in Supplementary Figure 6. For the models of Mental Health Care, Extramural Pharmaceutical Care and Other Non-Hospital Care, we relax the assumption that the errors are gamma-distributed, assuming a normal distribution instead. [↑](#footnote-ref-1)
