# Supplementary material for: Influence of changing patterns in lung cancer treatment and survival on the cost-effectiveness of CT screening: a modeling study
Source: eClinicalMedicine. 2025 Aug 29;88:103446. doi: 10.1016/j.eclinm.2025.103446 (PMC12418876; doi:10.1016/j.eclinm.2025.103446)
Supplement: LC_Treatment_Manuscript_clean_LBK (Page no: 22–26) [file mmc2.docx]

## Supplementary Tables and Figures

Supplementary Table 1 – Cost and Quality of Life Model Inputs

| **Type of cost/utility** | **Base value** | **Description** | **Index year** |
| --- | --- | --- | --- |
| ***Costs in EUR*** | | |  |
| Administrative overhead per participant | 7.96 | Subsidy per participant (16.74)^49^ in 2022 for the colorectal cancer screening programme in the Netherlands, less the cost of invitation (0.70) and the Fecal Immunochemical Test (8.08).^50^ | 2022 |
| Invitation costs | 0.70 | Invitation cost per screening event, in the 4-IN-THE-LUNG-RUN trial | 2022 |
| Risk Assessment | 4.60 | Cost of sending invitation survey and processing results, in the 4-IN-THE-LUNG-RUN trial. | 2022 |
|  |  |  |  |
| Initial LC care phase | 2,764-12,004 | Treatment costs are stratified by the stage (IA-IV), histology, and phase of care (first 6 months, intermittent, final 6 months), and the period (2013-2017 [little targeted or immunotherapy] and 2018-2021 [more widespread targeted or immunotherapy]). Stratified values are presented in Supplementary Table 4. | 2021 |
| Continuing LC care phase | 215-5,223 |  | 2021 |
| Terminal care – LC Death | 1816-7,164 |  | 2021 |
| Terminal care – other cause death | 1395-7,941 |  | 2021 |
| Terminal care in the absence of lung cancer | 1047.99 | Average health care costs in the final 6 months of life among those never observed to have lung cancer. | 2022 |
|  |  |  |  |
| Computed Tomography (CT) | 188 | CT cost per screening event and follow-up of false positive screen, using reference cost per National Health Care Institute. | 2022 |
| Possibly suspicious screening result | 188 | Additional CT screen. | 2022 |
| Suspicious screening result | 660 | Dutch Health Care Authority Unit cost for further investigation of a (suspected) malignancy in the respiratory organs. | 2022 |
|  |  |  |  |
| ***Health-Related Quality of Life*** | | | |
| Terminal LC | 0.59 | Final 6 months of LC. | - |
| Stage 1A-2 LC | 0.78 | Any life-year before terminal LC when diagnosed at stage  IA to II. | - |
| Stage 3A-4 LC | 0.69 | Any life-year before terminal LC when diagnosed at age IIIA to IV. | - |
| Male reference utility | 0.864-0.968 | The EQ5D derived reference utility values for the Netherlands from the EuroQoL group.^30^ Values are given by 10-year age category and sex. | - |
| Female reference utility | 0.746-0.910 |  | - |

*Supplementary Table 1 reports the health economic parameters applied to MISCAN model outputs to generate QALY gained and incremental costs of screening scenarios relative to no screening. Costs are applied for administration and invitation, for each screening event, for months spent in lung cancer care, and for follow-up of false positive results. Health-related quality of life is applied to all life years at a utility specific to the 10-year age group and sex, obtained from EuroQoL results. For those with lung cancer, a proportional disutility is assigned using the ratio of the lung cancer utilities to the assumed reference group of 65-year olds.^51^ Cost values presented here are scaled to 2023 price index level before being applied to simulated events.*

Supplementary Table 2 – First-line treatment regimen (in % of patient subgroup) by age category, stage of cancer and period of incidence

| Age-group: | [0, 65) | | | | [65, 75) | | | | [75,) | | | |
| --- | --- | --- | --- | --- | --- | --- | --- | --- | --- | --- | --- | --- |
| TNM Stage: | IA-II | | IIIA-IV | | IA-II | | IIIA-IV | | IA-II | | IIIA-IV | |
| Period of Incidence: | 2012-2017 | 2018-2021 | 2012-2017 | 2018-2021 | 2012-2017 | 2018-2021 | 2012-2017 | 2018-2021 | 2012-2017 | 2018-2021 | 2012-2017 | 2018-2021 |
| Targeted- or Immunotherapy Only | - | - | 4 | 13 | - | - | 3 | 11 | 0 | - | 3 | 8 |
| Chemo- and Targeted/Immuno therapy | - | - | 2 | 16 | - | - | 1 | 12 | - | - | 0 | 5 |
| Chemo-, Targeted/Immuno and Radiotherapy | - | 1 | 0 | 8 | - | 1 | 0 | 6 | - | 0 | - | 2 |
| Targeted/Immuno- and Radiotherapy | - | - | 0 | 1 | - | - | 0 | 0 | - | - | 0 | 0 |
| Chemotherapy | 1 | - | 36 | 16 | 1 | 1 | 31 | 15 | 1 | 0 | 14 | 8 |
| Chemo- and Radiotherapy | 5 | 3 | 24 | 14 | 5 | 3 | 19 | 12 | 3 | 2 | 8 | 6 |
| Radiotherapy | 22 | 29 | 3 | 2 | 34 | 41 | 5 | 3 | 51 | 55 | 9 | 7 |
| Surgery and Chemotherapy | 13 | 11 | 3 | 3 | 8 | 7 | 2 | 3 | 2 | 2 | 1 | 1 |
| Surgery | 52 | 50 | 2 | 2 | 44 | 40 | 2 | 2 | 22 | 21 | 1 | 2 |
| Other Combinations | 4 | 2 | 3 | 3 | 2 | 2 | 2 | 2 | 1 | 0 | 1 | 0 |
| None | 4 | 5 | 24 | 23 | 7 | 7 | 35 | 34 | 20 | 20 | 62 | 59 |
| N | 5,296 | 3,679 | 18,683 | 11,930 | 7,033 | 5,915 | 19,398 | 15,248 | 6,238 | 5,123 | 16,353 | 12,898 |

*Supplementary Table 2 presents the first-line treatment regimen of incident lung cancers, as recorded by the Netherlands Cancer Registry. The most common combinations of treatments are reported, with less frequent combinations absorbed in the category* *Other Combinations*. *For treatment regimens numbering fewer than 10 patients in each subgroup, the data is censored (shown as ‘-‘). Consequentially, the numbers reported reflect the percentage of patients relative to the total uncensored group.*

Supplementary Table 3 – Relative survival (%) at 3 years from diagnosis for lung cancer patients diagnosed between 2012-2021

|  |  | Females | | | | | | Males | | | | | |
| --- | --- | --- | --- | --- | --- | --- | --- | --- | --- | --- | --- | --- | --- |
|  |  | <65 | | 65-74 | | 75+ | | <65 | | 65-74 | | 75+ | |
| Cancer type | | 2013-2017 (ref.) | 2018-2021 (∆) | 2013-2017 (ref.) | 2018-2021 (∆) | 2013-2017 (ref.) | 2018-2021 (∆) | 2013-2017 (ref.) | 2018-2021 (∆) | 2013-2017 (ref.) | 2018-2021 (∆) | 2013-2017 (ref.) | 2018-2021 (∆) |
| Adeno-carcinoma | IA | 89 | +1 | 83 | +3 | 75 | +7 | 82 | +4 | 76 | +4 | 74 | +5 |
|  | IB | 84 | +4 | 79 | +3 | 63 | +8 | 80 | +4 | 74 | +5 | 65 | +9 |
|  | II | 73 | +7 | 61 | +13 | 53 | +9 | 66 | +11 | 55 | +11 | 44 | +12 |
|  | IIIA | 53 | +11 | 45 | +13 | 28 | +13 | 49 | +13 | 38 | +13 | 21 | +15 |
|  | IIIB | 38 | +15 | 27 | +13 | 13 | +12 | 28 | +19 | 27 | +7 | 13 | +8 |
|  | IV | 14 | +12 | 11 | +9 | 5 | +6 | 11 | +11 | 7 | +8 | 3 | +4 |
|  |  |  |  |  |  |  |  |  |  |  |  |  |  |
| Squamous-  cell Lung Cancer | IA | 81 | +5 | 74 | +2 | 74 | +1 | 83 | +4 | 76 | +2 | 70 | +3 |
|  | IB | 75 | +8 | 60 | +9 | 53 | +3 | 69 | +5 | 63 | +7 | 49 | +11 |
|  | II | 71 | +6 | 51 | +10 | 39 | +11 | 64 | +10 | 54 | +10 | 38 | +12 |
|  | IIIA | 44 | +11 | 29 | +12 | 18 | +12 | 40 | +12 | 32 | +12 | 20 | +10 |
|  | IIIB | 28 | +14 | 21 | +13 | 6 | +7 | 25 | +7 | 19 | +12 | 9 | +7 |
|  | IV | 7 | +6 | 5 | +5 | 3 | +4 | 7 | +5 | 6 | +4 | 3 | +4 |
|  |  |  |  |  |  |  |  |  |  |  |  |  |  |
| Other  NSCLC | IA | 83 | +1 | 70 | +5 | 67 | - | 75 | +2 | 66 | +3 | 60 | +4 |
|  | IB | 81 | +4 | 52 | +14 | 48 | +4 | 76 | +2 | 51 | +8 | 41 | +2 |
|  | II | 64 | +8 | 43 | +15 | 31 | +4 | 61 | +8 | 39 | +11 | 28 | +7 |
|  | IIIA | 42 | +13 | 33 | +5 | 11 | +2 | 29 | +13 | 24 | +5 | 13 | -1 |
|  | IIIB | 35 | +4 | 20 | +3 | 8 | +2 | 19 | +12 | 13 | +5 | 6 | +2 |
|  | IV | 10 | +10 | 5 | +4 | 3 | +1 | 8 | +5 | 3 | +3 | 2 | +1 |
|  |  |  |  |  |  |  |  |  |  |  |  |  |  |
| Small Cell Lung Cancer (SCLC) | IA | 56 | -6 | 56 | -6 | 56 | -6 | 48 | - | 48 | - | 48 | - |
|  | IB | 48 | +5 | 48 | +5 | 48 | +5 | 42 | +10 | 42 | +10 | 42 | +10 |
|  | II | 37 | +4 | 37 | +4 | 37 | +4 | 36 | +8 | 36 | +8 | 36 | +8 |
|  | IIIA | 40 | +6 | 33 | +1 | 16 | +7 | 35 | +10 | 21 | +12 | 14 | - |
|  | IIIB | 35 | +7 | 18 | +3 | 8 | +8 | 30 | +8 | 18 | +2 | 5 | +2 |
|  | IV | 7 | +1 | 3 | +1 | 0 | - | 4 | - | 2 | +1 | - | - |

*Supplementary Table 3 shows the relative survival of lung cancer patients diagnosed 2012-2021 in the Netherlands, stratified by the stage, histology and period of lung cancer diagnosis. Relative survival was estimated using the Ederer-II method, after which a Poisson curve was fitted using Generalized Linear Modelling to the monthly relative survival of each patient group (stratified by age at diagnosis [<65, 65-75, 75+], sex, stage of cancer [IA, IB, II, IIIA, IIIB, IV] and histology [Adenocarcinoma, Squamous Cell Lung Cancer, Other NSCLC, Small Cell Lung Cancer]). Period-specific estimates were generated by allocating the person-years of lung cancer patients diagnosed [2012, 2021] to separate 2013-2017 and 2018-2021 periods. The relative survival point estimates is shown for the 2013-2017 period. For the 2018-2021 period the increase in percentage points is shown relative to the 2013-2017 period.*

Supplementary Table 4 – Cost estimates per patient-month of care, stratified by stage, histology and phase of treatment

|  |  | Adeno Carcinoma | | Squamous Cell Lung Cancer | | Other Non-Small Cell Lung Cancer | | Small Cell Lung Cancer | |
| --- | --- | --- | --- | --- | --- | --- | --- | --- | --- |
| Expenditure  Category | Cancer  Stage | 2013-2017 (€) | 2018-2021  (∆ in €) | 2013-2017 (€) | 2018-2021  (∆ in €) | 2013-2017 (€) | 2018-2021  (∆ in €) | 2013-2017 (€) | 2018-2021  (∆ in €) |
| Initial Care  By Month | IA | 3,468 | 259 | 3,728 | 130 | 2,764 | 62 | 5,855 | -542 |
|  | IB | 3,699 | 750 | 4,107 | -300 | 3,201 | 466 | 5,724 | 922 |
|  | II | 5,388 | 146 | 5,957 | -425 | 3,943 | 313 | 7,651 | 618 |
|  | IIIA | 6,365 | 1,639 | 6,492 | 713 | 4,671 | 1,212 | 8,393 | 215 |
|  | IIIB | 6,773 | 2,669 | 6,314 | 1,923 | 5,686 | 1,756 | 9,342 | -670 |
|  | IV | 6,750 | 5,254 | 5,301 | 3,448 | 5,010 | 3,394 | 8,048 | -574 |
|  |  |  |  |  |  |  |  |  |  |
| Continuing  Care  By Month | IA | 215 | 244 | 378 | 97 | 316 | 126 | 506 | 75 |
|  | IB | 296 | 267 | 365 | 171 | 315 | 162 | 352 | 186 |
|  | II | 428 | 526 | 422 | 271 | 351 | 257 | 360 | 248 |
|  | IIIA | 640 | 969 | 536 | 758 | 590 | 823 | 475 | 351 |
|  | IIIB | 1,111 | 1,799 | 997 | 1,284 | 761 | 1,245 | 571 | 231 |
|  | IV | 2,130 | 3,093 | 1,615 | 2,376 | 1,226 | 2,506 | 1,013 | 358 |
|  |  |  |  |  |  |  |  |  |  |
| Terminal  (LC) Care  By Month | IA | 3,200 | -540 | 3,144 | -1,028 | 2,075 | -259 | 2,235 | 1,407 |
|  | IB | 3,304 | -339 | 2,654 | 66 | 2,463 | 51 | 6,457 | -3,641 |
|  | II | 4,097 | -749 | 4,064 | -505 | 2,604 | -357 | 2,629 | 403 |
|  | IIIA | 3,983 | -27 | 3,965 | -215 | 3,046 | 532 | 3,882 | -307 |
|  | IIIB | 4,370 | 834 | 3,349 | 1,080 | 4,086 | -244 | 3,936 | 68 |
|  | IV | 5,409 | 1,754 | 4,011 | 1,240 | 4,350 | 1,390 | 4,737 | 539 |
|  |  |  |  |  |  |  |  |  |  |
| Terminal  (OC) Care  By Month | IA | 3,535 | -1,192 | 3,677 | -1,663 | 2,100 | -515 | 7,941 | -6,546 |
|  | IB | 3,459 | -1,137 | 3,411 | -1,418 | 2,173 | 2 | 2,573 | -713 |
|  | II | 3,760 | -628 | 4,677 | -1,832 | 3,017 | -1,289 | 4,342 | 229 |
|  | IIIA | 4,244 | -672 | 4,066 | -917 | 3,458 | -1,409 | 3,690 | -1,366 |
|  | IIIB | 3,800 | 398 | 3,620 | 499 | 4,081 | -409 | 3,911 | -351 |
|  | IV | 4,360 | 2,021 | 3,373 | 751 | 4,048 | 319 | 4,872 | -426 |

*Supplementary Table 4 shows the monthly treatment costs of lung cancer patients diagnosed 2012-2021 in the Netherlands, stratified by the stage, histology and period of lung cancer diagnosis. Estimates are obtained using regression modelling of medical expenditures of individual-level claims data using a nationwide (n=19.2m) dataset, linked to cancer registry data of lung cancer incidence and tumor characteristics (n=137,129). The terminal care (LC) phase represents the patient-month cost of the final 6 months of life for patients recorded to die of lung cancer, for the (OC) cases it represents patient-month cost for those reported to die of other causes. The initial phase represents the first 6 months of treatment. The continuing phase represents any intermittent care between the initial and terminal phase, up to 5 years. Exact methods, as well as estimates by histology, sex and treatment regimen are reported in our previous publication.^7^ Cost values here are shown in their original 2021 price index level. For application to the expected cost of treatment in MISCAN simulations, values are scaled to the 2023 price index level.*
